# Physico-Chemical Characterization and Biopharmaceutical Evaluation of Lipid-Poloxamer-Based Organogels for Curcumin Skin Delivery

**DOI:** 10.3389/fphar.2019.01006

**Published:** 2019-09-12

**Authors:** Aryane Alves Vigato, Samyr Machado Querobino, Naially Cardoso de Faria, Ana Carolina Bolela Bovo Candido, Lizandra Guidi Magalhães, Cíntia Maria Saia Cereda, Giovana Radomille Tófoli, Estefânia Vangelie Ramos Campos, Ian Pompermayer Machado, Leonardo Fernandes Fraceto, Mirela Inês de Sairre, Daniele Ribeiro de Araujo

**Affiliations:** ^1^Human and Natural Sciences Center, ABC Federal University, Santo André, Brazil; ^2^Department of Biomedical Sciences, State University of Minas Gerais, Passos, Brazil; ^3^Research Group on Natural Products, Center for Research in Sciences and Technology, University of Franca, Franca, Brazil; ^4^São Leopoldo Mandic Research Unit, São Leopoldo Mandic Faculty, Campinas, Brazil; ^5^Department of Environmental Engineering, State University “Júlio de Mesquita Filho”, Sorocaba, Brazil; ^6^Department of Fundamental Chemistry, Institute of Chemistry, University of São Paulo, São Paulo, Brazil

**Keywords:** organogel, pluronic, skin-delivery, curcumin, oleic acid

## Abstract

Organogels (ORGs) are semi-solid materials, in which an organic phase is immobilized by a three-dimensional network composed of self-organized system, forming the aqueous phase. In this context, lipid–Pluronics (PLs) ORGs form a two-phase system which can be effectively used as skin delivery systems, favoring their permeation across the skin. In this study, we presented the development of ORG skin drug-delivery systems for curcumin (CUR), a liposoluble phenolic pigment extracted from the turmeric rhizome. In special, we designed the formulation compositions in order to carry high amounts of CUR soluble in oleic acid (OA), as organic phase, entrapped into an aqueous phase composed of micellar PL-based hydrogels by associating two polymers with different hydrophilic–lipophilic balances, Pluronic F-127 (PL F-127), and Pluronic L-81 (PL L-81), to enhance the permeation across the skin. Results revealed that the incorporation of PL L-81 favored the CUR incorporation into micelle–micelle interface. CUR insertion into OA-PL F-127/L-81 reduced both G’/G” relationship (∼16 x) and viscosity values (η* ∼ 54 mPa.s, at 32.5°C), disturbing the ORG network structural organization. *In vitro* permeation assays through Strat-M^®^ skin-model membranes showed that higher CUR-permeated amounts were obtained for OA-PL F-127/L-81 (4.83 µg.cm^−2^) compared to OA-PL F-127 (3.51 μg.cm^−2^) and OA (2.25 μg.cm^−2^) or hydrogels (∼1.2 μg.cm^−2^, p < 0.001). Additionally, ORG formulations presented low cytotoxic effects and evoked pronounced antileishmanial activity (IC_50_ < 1.25 µg.ml^−1^), suggesting their potential use as skin delivery systems against *Leishmania amazonensis*. Results from this study pointed out OA-PL-based ORGs as promising new formulations for possible CUR topical administration.

## Introduction

Curcumin (CUR) and its derivatives have shown a wide variety of biological activities, such as anti-oxidant (Dall’Acqua et al., 2016), anti-inflammatory (Zhu et al., 2016), anti-tumor (Han et al., 2011), antimicrobial (Cetin-Karaca and Newman, 2015), and antiparasitic effects (Morais et al., 2013), as well as for the treatment of ulcers (Magalhaes et al., 2009) and skin diseases (Patel et al., 2009; Rachmawati et al., 2015), among others (Aggarwal and Harikumar, 2009). Despite its efficacy and safety, CUR has not yet been approved as a therapeutic agent (Anand et al., 2008). In addition, due to its physico-chemical limitations such as low aqueous solubility and low bioavailability (Priyadarsini, 2014), several studies have been devoted to developing new pharmaceutical formulations to overcome those limitations. In fact, CUR extensive first-pass biotransformation and low aqueous solubility became an interesting molecule for skin delivery (Anand et al., 2008).

In this context, skin delivery is an important strategy for drug administration, since this procedure is non-invasive and avoids first-pass biotransformation and enables the use of self-administered pharmaceutical forms, improving patient compliance. However, the clinical efficacy of this type of administration depends on the drug physico-chemical and pharmacological properties, as well as its bioavailability at the site of action, which is limited by the low permeability of the stratum corneum (Godin and Touitou, 2007; Prausnit and Langer, 2008). Considering that most skin pathological processes occur locally, CUR topical application may offer the advantage for delivering the molecule into the site of action. Nanocarrier systems such as gels and nanoemulsions can therefore provide the chemical stabilization and permeation of the CUR molecule (Rachmawati et al., 2015).

Among the various nanostructured systems are those formed by the poloxamers or Pluronics^®^ (PL), copolymers used in preformulations such as hydrogels and as aqueous phase of organogels (ORGs). Particularly, as recent hybrid systems, ORGs stand out as semi-solid colloidal systems that has an oil phase dispersed in an aqueous phase, being used as reservoirs for lipophilic molecules. Those systems present advantages over conventional formulations (creams, ointments, hydrogels) due to their ability to incorporate higher concentrations of lipophilic molecules (Patel et al., 2009; Esposito et al., 2018) such as CUR, being capable to modulate the time and rate of skin permeation according to their composition. In fact, lipid (oil or organic phase) and PL (aqueous phase) gels form a two-phase system which can be effectively used as skin delivery promoters for hydrophilic and lipophilic drugs, favoring their permeation across the stratum corneum. Additionally, ORGs present other advantages such as (i) adhesion to the skin due to the formation of a homogeneous film on the stratum corneum surface, (ii) increases the contact area with the application site, (iii) improves the chemical stability of incorporated molecules, and (iv) absence of organic solvents in formulation preparation, which increases the ORG biocompatibility (Vintiloiu and Leroux, 2008; Iwanaga et al., 2012; Esposito et al., 2018).

In this study, we have developed ORGs with organic phase (OP) composed of oleic acid (OA), a free fatty acid (C_18_H_34_O_2_) well-described as permeation enhancer, incorporated into an aqueous phase (AP) containing Pluronic F-127 (PL F-127) isolated or in association with Pluronic L81 (PL L-81). The PL physico-chemical features such as molecular weight (PL F-127 = 12,400 g.mol^−1^ and PL L-81 = 2,800 g.mol^−1^), hydrophilic–lipophilic balances (HLB, PL F-127 = 22 and PL L-81 = 2), and polypropylene oxide (PPO):polyethylene oxide (PEO) relationships (PL F-127 = ∼1:3 PPO : PEO and for PL L-81 = ∼7:1 PPO : PEO) provide a differential structural organization (Oshiro et al., 2014) to modulate the CUR permeation. Then, we have studied those ORG systems regarding to their physico-chemical, structural, and biopharmaceutical properties e.g., the micellization process, the sol–gel transition, rheological features, structural organization and, especially, the influence of OA-PL F-127/L-81 association on CUR permeation profile, photostability, citotoxicty, and its biological activity.

## Materials and Methods

### Chemicals and Reagents

Pluronic^®^ F-127 (PL F-127), Pluronic^®^ L-81 (PL L-81), OA, and CUR were purchased from Sigma–Aldrich (St. Louis, MO, USA). All chemicals and solvents were analytical grade.

### High-Performance Liquid Chromatography (HPLC) Analysis

CUR analysis was performed using an HPLC system (Ultimate 3000, Chromeleon 7.2 software, Thermo Fisher Scientific, Waltham, USA) with a gradient pump, DAD detector, and C18 column (150 x 4.6 mm, 5 µm; Phenomenex). Drug samples were analyzed at 425 nm, 0.8 ml/min flow rate at 25°C. The mobile phase was composed by a mixture of acetonitrile and water with acetic acid (0.05%) solution (70:30). The drug retention time was 3.4 min. All results represent three experiments, in 3 days, performed in triplicate. The limits of detection (LD) and quantification (LQ) were determined from a standard curve of CUR at 10, 20, 40, 60, 80, and 100 µg/ml. The LD and LQ values were 0.31 and 0.94 µg/ml, respectively. CUR concentration was determined using the equation y = 2.616x ± 0.003 with correlation coefficient (R²) value of 0.998.

### ORG Preparation

Initially, the AP was prepared by mixing appropriate amounts of PL F-127 (20% wt) isolated or in association with PL L-81 (0.6%) in HPLC-grade water under ice bath with continuous stirring (350 rpm, for at least 12 h) until the solution became transparent. The hydrogels formulations, used in this study as AP, were previously designed and characterized (Oshiro et al., 2014). For OP preparation, CUR (2 mg) was added to 2 ml of OA under magnetic stirring (350 rpm, at 25°C) until the complete drug dissolution. Then, OP was added to the AP (1:4 v/v) and magnetically stirred (350 rpm) until the formation of an homogeneous gel (Boddu et al., 2014; Vigato et al., 2019). Finally, sodium benzoate (0.25% wt) was added to all formulations as a preservative. For comparisons regarding to morphology and permeation profiles, hydrogels were prepared at the same composition from ORG AP. All formulation compositions are presented on **Table 1**.

**Table 1:** Formulations components for different ORG containing curcumin (CUR).

Formulations	Organic phase(OP)	Aqueous phase(AP)	
	Oleic acid (OA) organic solvent	Pluronic F-127(F-127, %wt)	Pluronic L-81(L-81, %wt)
OA-F-127	OA	20	–
OA-F-127—CUR		20	–
OA-F-127/L-81		20	0.6
OA-F-127/L-81—CUR		20	0.6
			
F-127-H	–	20	–
F-127-CUR-H	–	20	–
F-127/L-81-H	–	20	0.6
F-127/L-81-CUR-H	–	20	0.6

Organic: aqueous phase ratio of 1:4 (OP : AP, v/v); CUR final concentration was 0.1%. H—indicates hydrogels formulations (without oleic acid); OA, oleic acid; PL F-127, Pluronic^®^ F-127; PL L-81, Pluronic^®^ L-81; CUR, curcumin.

### Organoleptic Characterization, Drug Content, and pH Determination

The ORG formulations were evaluated by color, odor, and phase separation. The pH measurements were performed for all ORG formulations inserting the probe into the ORGs until the equilibrium determination. For drug content determination, samples of ORGs (0.05 g) were weighted, mixed with 10 ml of acetonitrile:water (70:30 v/v) and sonicated for ∼ 20 min. Samples of 1 ml were filtered (nylon syringe filter, 0.22-µm pore) and analyzed by HPLC for determining CUR concentration. Drug content was expressed as a percentage.

### Structural and Morphological Analysis

ORG formulations were analyzed by atomic force microscopy (AFM). For samples preparation, a thin film of each formulation was disposed in a glass slide and dried and dripped on a silicon plate. Samples were analyzed in a Nanosurf easyScan 2 Basic microscope (Nanosurf, Switzerland) in non-contact mode in an instrument equipped with a TapAl-G cantilever (BudgetSensors, Bulgaria) operated at a scan rate of 90Hz. Images (256×256 pixels, TIFF format) were captured in time mode and were analyzed using Gwyddion software. In addition, the formulation structural properties were analyzed by X-ray diffraction (XRD) technique, using a Rigaku Miniflex II instrument with CuKα1 radiation (λ = 1.5406 Å). ORG samples were pressed against a glass sample holder to obtain a homogeneous surface and analyzed over the 5–70° 2θ range, employing a 0.05° step with 1 s of integration time.

### Differential Scanning Calorimetry (DSC) and Rheological Analysis

Differential scanning calorimetry (DSC) experiments were carried out by a Netzsch DSC Polyma Calorimeter (NETZSCH, Selb, Germany). ORG samples (20 mg) were placed in a sealed aluminum pan and analyzed by three cycles (heating-cooling-heating) from 0 to 50°C at 5°C/min rate. Thermograms were presented as heat flux (J/g) against temperature (°C). For all analyzes, an empty pan was used as the reference.

For rheological analyses, an oscillatory Kinexus rheometer (Malvern Instruments Ltd., UK) with cone-plate geometry was employed. In order to determine the sol–gel transition temperature (T_sol–gel_), the frequency was set at 1 Hz, and a temperature range from 10 to 50°C was used. Additionally, for frequency sweep mode, the temperature was kept at 32.5°C, and formulations were analyzed from 0.1 to 10 Hz. For both measurements, the oscillatory mode was used to obtain the elastic (G’) and viscous modulii (G”), as well as viscosity (η*) values for each formulation. Data were analyzed with the RSpace for Kinexus^®^ software.

### *In Vitro* Permeation Studies

For *in vitro* permeation assays, vertical Franz-type diffusion cells (Vision Microette Plus; Hanson Research, Chatsworth, CA, USA) were used. The cells presented two compartments, donor (1.72 cm^2^ permeation area) and receptor (7 ml), separated by an artificial skin-model membrane (Strat-M^®^ membranes, 25-mm discs, Millipore Co., USA, ultrafiltration membrane, 325 µm thick) (Uchida et al., 2015; Kaur et al., 2018). Each formulation (0.3 g/cm^2^) was applied to the donor compartment (in contact with the upper surface of the artificial membrane). The receptor compartment was filled with 7 ml of pH 7.4 sodium phosphate (5 mM) with sodium chloride (154 mM) buffer and magnetically stirred (350 rpm) at 32.5 ± 0.5°C for 48 h. During the time interval from 15 min to 48 h, aliquots (1 ml) from the receptor compartment were collected and analyzed by HPLC. All experiments were performed in triplicate. The cumulative amounts of permeated CUR were expressed as µg.cm^−2^, and the results were plotted as a function of time (h). For data analyzes, flux values were obtained from the slope of the curve over the 8-h period. Data were analyzed according to the equation (eq. 1):

(1) J = P. Cd

where J (µg.cm^−2^.h^−1^) is the drug flux across the membrane, P (cm.h^−1^) is the permeability coefficient, and Cd (µg.cm^−3^) is the drug concentration in the donor compartment. The lag time was calculated by extrapolating a straight line to time axis (de Araujo et al., 2010).

### *In Vitro* Cytotoxicity and Antileishmanial Activity

Epidermal keratinocytes (HaCaT cell line, Thermo Fisher Scientific, Waltham, Massachusetts, USA) were used for the cytotoxicity experiments. Cells were seeded for 48 h in 96-well plates (2.104 cells/well), in Dubelcco’s Modified Eagle Medium (DMEM; Gibco Laboratories, Grand Island, NY, USA) with 10% (v/v) fetal bovine serum (pH 7.2–7.4), humidified atmosphere at 37°C and 5% CO_2_) and 100 μg.ml^−1^ of penicillin/streptomycin. For experiment design, ORG formulations were previously diluted in DMEM medium on concentration range from 10 to 100 mg.ml^−1^, and 200 µl from each solution were used for cell treatment during 24 h. Then, 100 µl of MTT solution (5 mg/ml, in phosphate buffered saline) was added to each well and incubated with cells for 4 h. After that, MTT solution was removed and 50 μl of DMSO added to the wells for 10 min. Absorbance was measured at 570 nm. For comparisons with non-toxicity, cells were treated only with DMEM at the same volume used for ORGs.

For pharmacological assays, in order to evaluate the antileishmanial activity, *Leishmania amazonensis* promastigote forms (MHOM/BR/PH8) were maintained in RPMI 1640 (Gibco) culture medium supplemented with 10% fetal bovine serum, penicillin (100 UI/ml), and streptomycin (100 μg/ml). Subsequently, about 1 x 10^6^ parasites were seemed in 96-well plates, and ORGs dissolved in culture medium were added at concentrations from 1.25 to 10 μg.ml^−1^ to the cultures. Amphotericin B (Sigma Aldrich Chem. Co., 97% purity) was added to cultures at concentrations ranging from 0.05 to 0.40 μg/ml and used as positive control. CUR was dissolved in dimethyl sulfoxide (DMSO) and added at same concentrations described before. Cultures were incubated at 25°C for 24 h, and the antileishmanial activity was determined by verifying the growth of the promastigote forms was inhibited, as revealed by counting the total number of live promastigotes using a Neubauer chamber according to the flagellar motility. The results were expressed as the mean of the percentage of growth inhibition relative to the negative control (RPMI 1640 medium+0.1% DMSO or RPMI 1640 medium). Two experiments were performed in triplicate. The 50% inhibitory concentration (IC_50_) values were determined by means of non-linear regression curves using GraphPad Prism version 5.0 software for Windows (GraphPad software, USA) (Bezerra et al., 2006).

### Statistical Analysis

Results were presented as mean ± standard deviation. For statistical comparisons, one-way analysis of variance (ANOVA) with Tukey–Kramer *post hoc* test was used. Statistical significance was defined as p < 0.05.

## Results

### Structural and Morphological Characterization, pH, and Drug Content Determination

**Figure 1:**
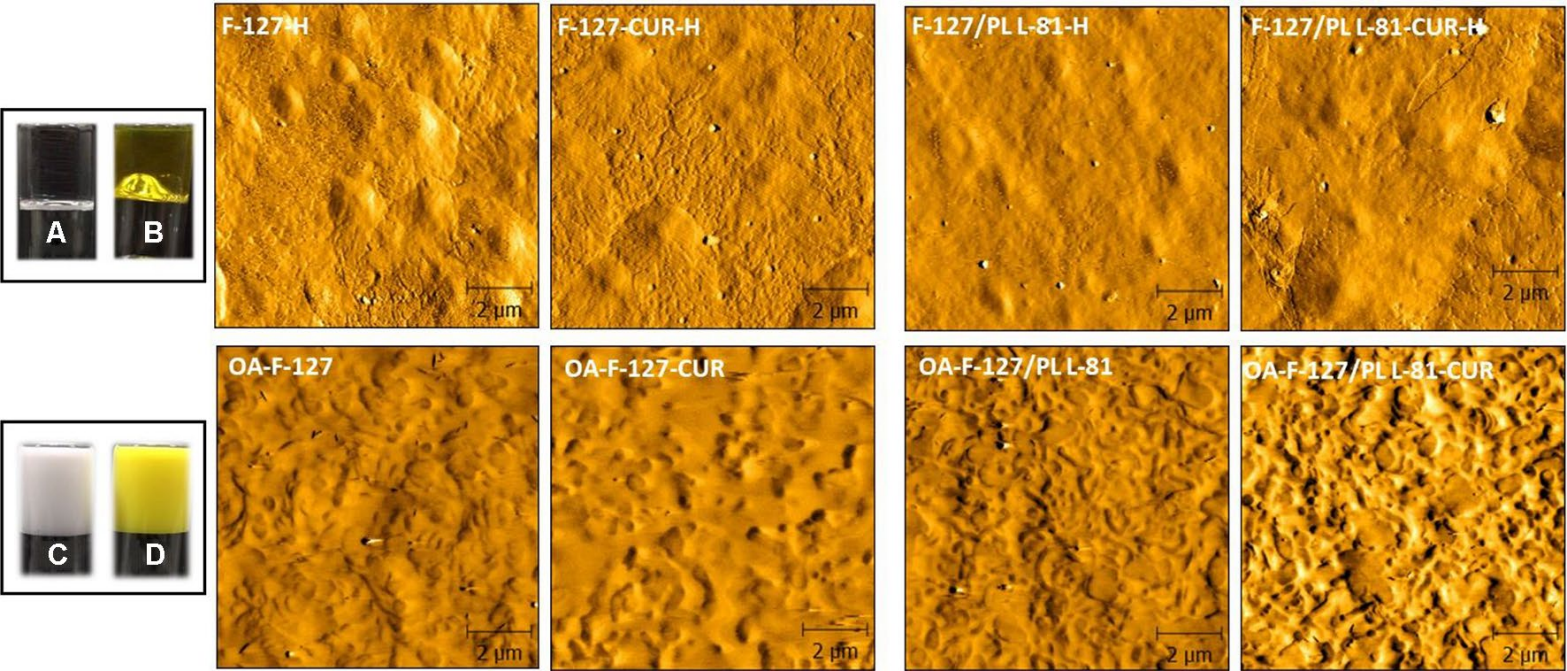
Micrographs for organogels and hydrogel formulations obtained from atomic force microscopy (AFM). **(A)** F-127/L-81-H, **(B)** F-127/L-81-CUR-H, **(C)** OA-F-127/L-81, and **(D)** OA-F-127/L-81-CUR. H—indicates hydrogel formulations (without oleic acid). OA, oleic acid; F-127, Pluronic^®^ F-127; L-81, Pluronic^®^ L-81; CUR, curcumin.

**Figure 2:**
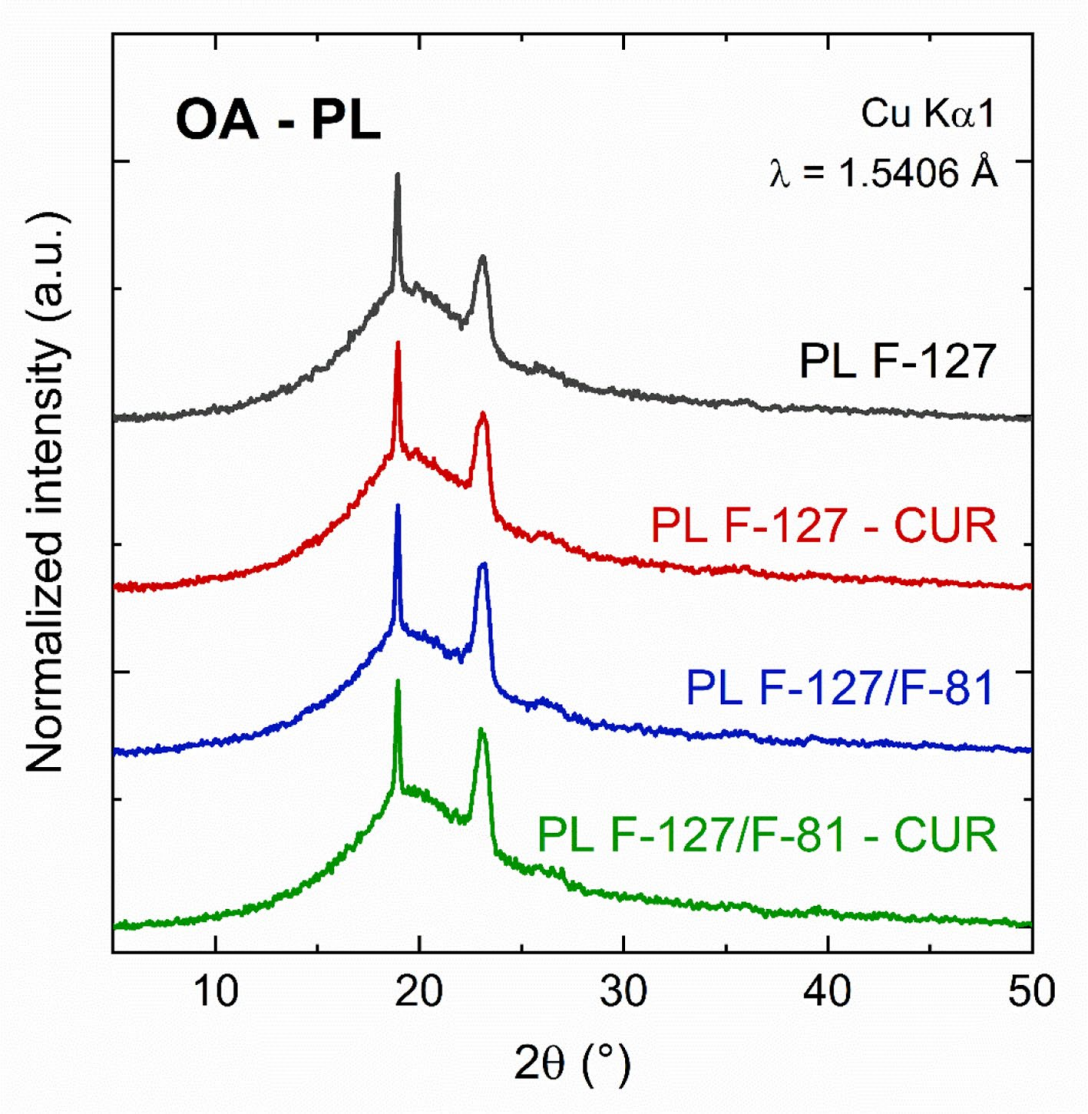
X-ray diffraction patterns for the ORG formulations OA-PL F-127, OA-PL F-127-CUR, OA-PL F-127/L-81, and OA-PL F-127/L-81-CUR (OA, oleic acid; PL F-127, Pluronic^®^ F-127; PL L-81, Pluronic^®^ L-81; and CUR, curcumin.

Before CUR incorporation, ORGs presented white-opaque aspect while hydrogels were colorless and transparent, as observed on **Figures 1 A, C**. After CUR incorporation, all formulations became yellow (opaque or clear, **Figures 1 B, D**), and neither particulate materials nor any phase separation were observed. For morphological characterization, AFM images revealed smooth surfaces with small and sparse protuberances for hydrogels (control formulations), as a result of the dry process before analysis. On the other hand, ORG morphology was characterized by wrinkles distributed for all surface, which can be attributed to the incorporation of the OP into the hydrogels forming a system with low water content resulting in a different morphology from hydrogels. Morphological differences between hydrogels and ORGs were also previously described using scanning electron microscopy (SEM) (Vigato et al., 2019). The ORGs were also characterized by X-ray diffraction (XRD), as shown in **Figure 2**. All formulations exhibited the same pattern, with two diffraction peaks at 19 and 23°, which are correspondent to the PL F-127 and crystalline structure (Shin et al., 2000; Saxena et al., 2012). The very broad diffraction peak observed in the diffractograms indicates that the ORGs also exhibit an amorphous character. This feature can be attributed to the presence of OA as OP, which disturbs the well-known crystalline nature of PL after incorporation into the formulations.

The pH values for ORG formulations were from 5.5 to 5.9, for OA-PL F-127 and OA-PL F-127/L-81, even after CUR incorporation. Those results are in agreement with previous reports about other ORG compositions such as lanolin-PL F-127 (Vigato et al., 2019), ricinoleic acid-PL F-127 (Boddu et al., 2015), and lecithin-PL F-127 (Agrawal et al., 2010), reflecting no possible risk of skin irritation. For all formulations, the CUR content was ∼ 95.2%, confirming the homogeneous drug distribution throughout the ORGs.

### Differential Scanning Calorimetry (DSC) and Rheological Analysis

**Figure 3:**
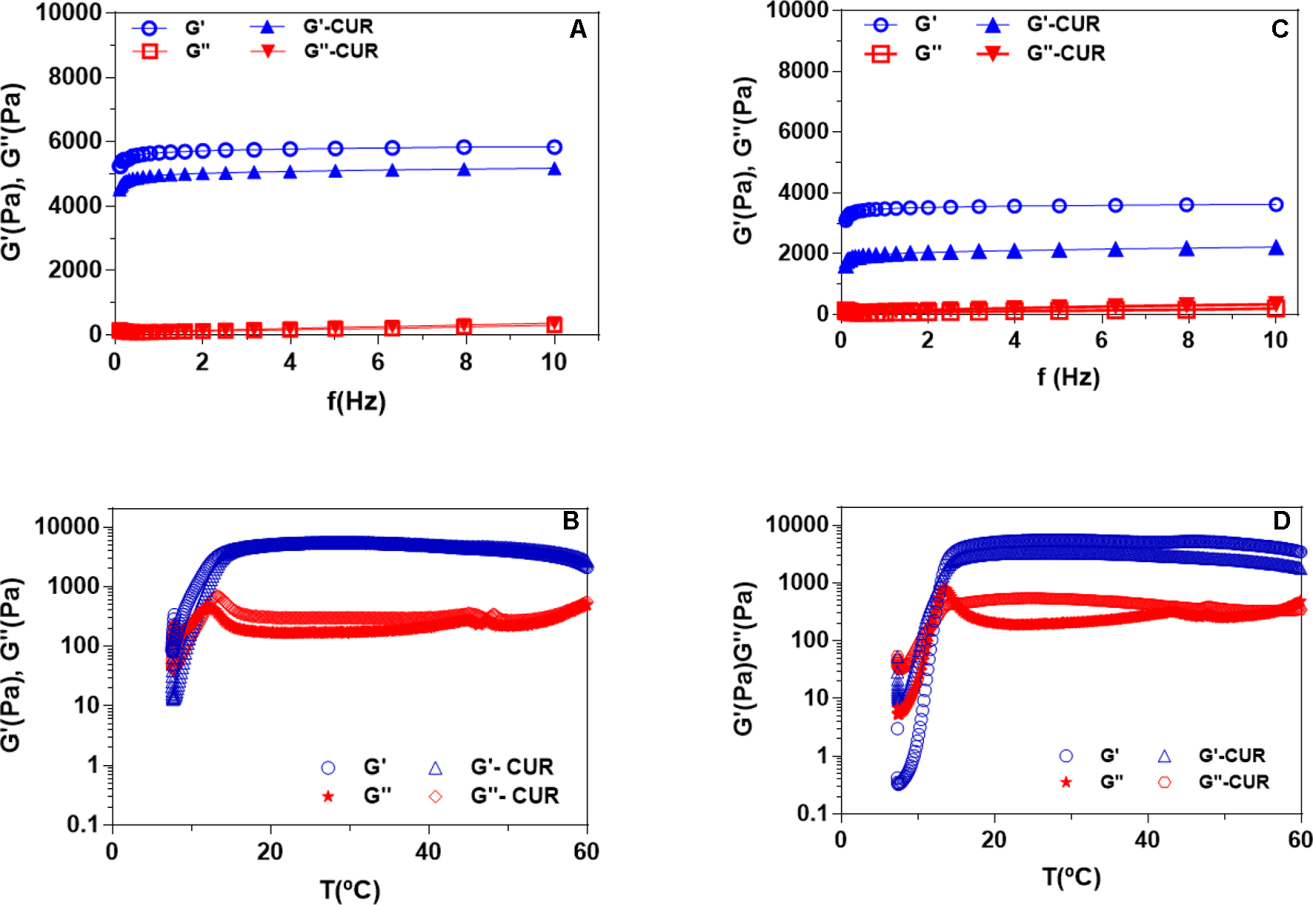
Rheograms display the frequency sweep **(A**, **C)** and sol–gel transition **(B**, **D)** analysis for organogels composed of OA-F-127 **(A**, **B)** OA-F-127/L-81 **(C**, **D)**.

All ORG formulations were analyzed regarding to micellization and sol–gel transition processes considering their initial (T_onset_), peak (T_peak_), final (T_endset_) phase transition temperatures; enthalpy change (ΔH); and rheological parameters such as elastic (G’) and viscous (G”) moduli, as well as viscosity (η*). All results are presented on **Table 2** and **Figure 3**.

**Table 2:** Temperatures (T), enthalpy variation (ΔH), and rheological parameters relative to the organogels phase transition before and after curcumin incorporation.

Formulations	DSC				Rheology				
	T_onset_ (°C)	T_peak_ (°C)	T_endset_ (°C)	ΔH_m_ (J.g^−1^)	G’(Pa)	G’’ (Pa)	G’/G’’	η*(32.5°C)(x 10³, mPas.s)	Tsol–gel(°C)
**OA-F-127**	10.2	12.0	14.2	0.44	5,666	94.1	60.2	84.6	14.8
**OA-F-127-CUR**	10.1	12.9	15.3	3.34	4,960	111.3	44.6	85.5	15.9
**OA-F-127/L-81**	10.1	11.7	12.8	0.36	3,481	63.7	54.6	85.9	15.3
**OA-F-127/L-81-CUR**	10.5	12.3	12.7	0.52	1,984	124.3	15.9	53.8	15.2

T_onset_, T_peak_, and T_endset_ represent the initial, peak, and final temperatures for phase transitions. G’ (elastic) and G” (viscous) moduli, apparent viscosity (η*), and sol–gel transition temperatures (Tsol-gel). OA, oleic acid; F-127, Pluronic^®^ F-127; L-81, Pluronic^®^ L-81; CUR, curcumin.

DSC analysis revealed that, in general, T_onset_ and T_peak_ values were similar for all formulations, but small shifts were observed on phase transition temperatures in response to CUR and/or PL L-81, since T_endset_ values were reduced from 14.2 to 12.8°C after PL L-81 incorporation, while CUR insertion increased the T_endset_ value for 15.3°C, being observed only for OA-PL F-127/L-81. Regarding to enthalpy variation, a more pronounced CUR interference was observed for the systems composed of OA-PL F-127, since similar ΔH values were obtained before and after CUR incorporation into the OA-PL F-127/L-81. Those results reflect the CUR influence on the phase transition process, considering the possible drug dispersion into the ORG oil phase and the potential hydrophobic interactions between the OA carbon backbone (C18) and CUR.

The rheological parameters elastic (G’) and viscous (G”) moduli, as well as apparent viscosity (η*), were determined for all ORG formulations. Additionally, the sol–gel transition temperature was also obtained in order to predict the possible influence of CUR incorporation and both PL on ORG AP composition, their compatibility and structural organization. In this context, ORG formulations were also analyzed specially according to the PL types forming binary systems into the AP (**Table 2** and **Figure 3**).

Rheological analysis revealed similar T_sol–gel_ values ranging from 14.8 to 15.9°C for isolated PL F-127 and its binary system with PL L-81, as well as after CUR incorporation. All ORG formulations presented viscoelastic behavior, being stable under temperature variation, since G’ > G” values. However, the presence of CUR reduced the G’/G” relationships (from ∼ 60 to 16), specially for the system OA-PL F-127/L-81. Similar effects were also observed on η* parameter (**Table 2**), which can be attributed to the influence of CUR molecules into the gels three-dimensional network formed by the AP, disturbing their structural organization (Mady et al., 2016).

Regarding to the frequency sweep analysis, results revealed that the parameters G’ and G” were not significantly affected by the applied frequency range, since G’ > G” were observed for all formulations. After CUR incorporation, the system OA-PL F-127/L-81 presented the lowest G’/G” relationship value, compared to OA-PL F-127, showing the drug influence on ORG structural organization in addition to the presence of L-81 into the AP, as described before for other parameters such as viscosity and T_sol–gel_.

### *In Vitro* Permeation Studies

**Figure 4:**
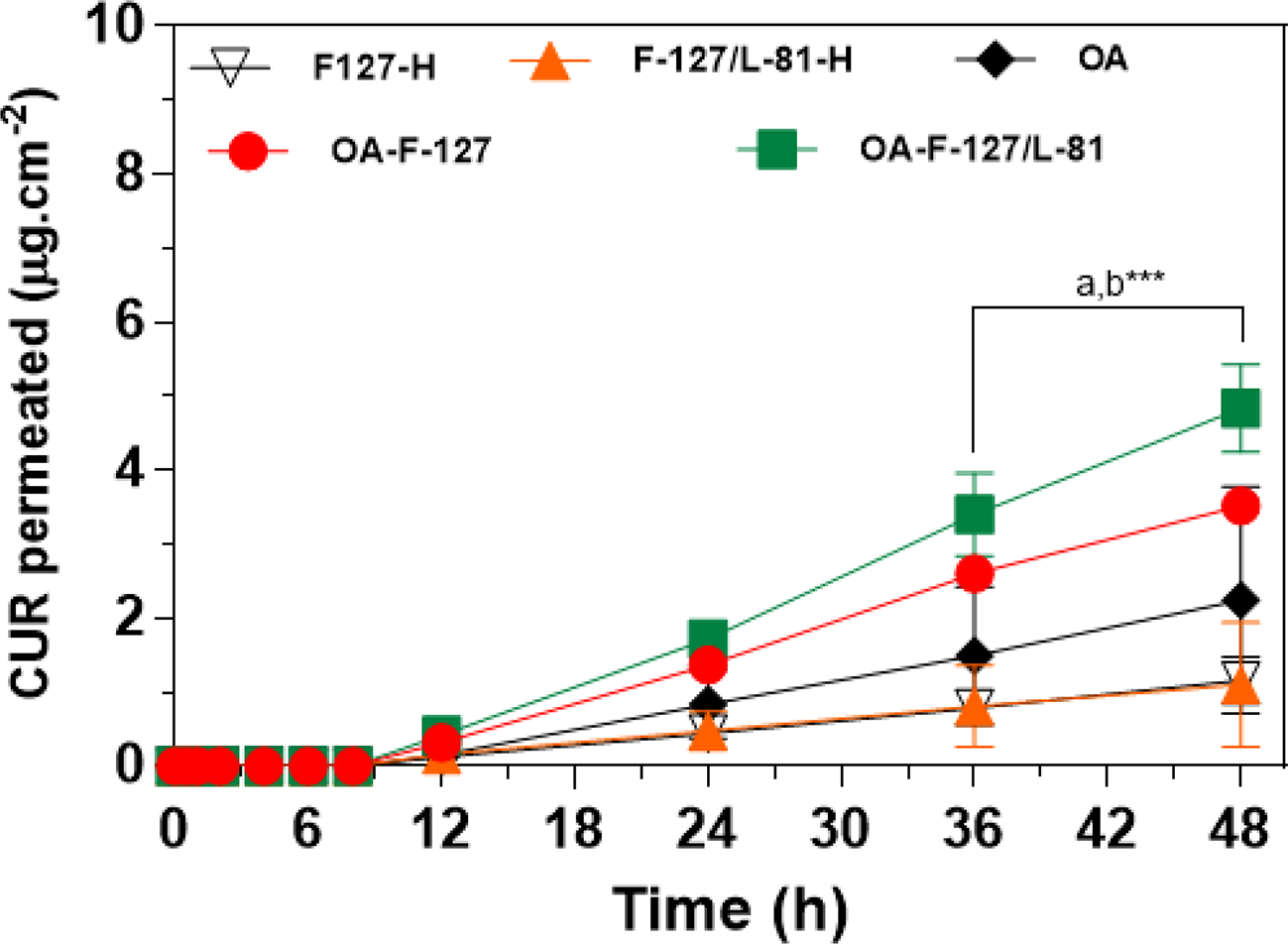
Curcumin (CUR) permeation profiles from formulations across Strat-M^®^ artificial membranes (mean ± standard deviation, n = 4–6). Organogels formulations containing 0.1% CUR. OA, oleic acid; PL F-127, Pluronic^®^ F-127; PL L-81, Pluronic^®^ L-81; CUR, curcumin. H—indicates hydrogels formulations (without oleic acid). a- OA-F-127/L-81 *vs.* F-127/L-81-H (PL-F-127-H; b- OA-F-127/L-81 *vs.* OA-F-127. ***p < 0.001.

In order to characterize the CUR permeation from ORG formulations, experiments were performed using Strat-M^®^ artificial membranes as barrier. In addition, hydrogels composed of each AP used for preparing the ORGs were also included as formulations, for evaluating the influence of OA- and PL-type on CUR permeation profiles. Results from those assays are summarized on **Figure 4** and **Table 3**.

**Table 3:** Curcumin permeation parameters across Strat-M^®^ membranes from organogel formulations.

Formulations	Flux (µg.cm^−2^.h^−1^)	Permeability coefficient (cm.h^−1^)	Lag time (h)
OA	0.047 ± 0.003	0.028 ± 0.002	3.66 ± 0.21
OA-F-127	0.076 ± 0.005	0.107 ± 0.001	3.49 ± 0.18
OA-F-127/L-81	0.102 ± 0.006^a***,b**^	0.170 ± 0.003 ^a***,b***^	3.61 ± 0.22
F-127-H	0.024 ± 0.001	0.040 ± 0.002	3.14 ± 0.12
F-127/L-81-H	0.025 ± 0.001	0.041 ± 0.004	3.44 ± 0.19

F-127, Pluronic^®^ F-127; L-81, Pluronic^®^ L-81; OA, oleic acid; H—indicates hydrogel formulations (without oleic acid). Data presented as mean ± SD, Statistical differences are expressed as: a- OA-PL F-127/L-81 vs. hydrogels (F-127/L-81-H and F-127-H); b- OA-F-127/L-81 vs. OA or OA-F-127. ***p < 0.001 and **p < 0.01.

Results from permeation experiments across Strat-M^®^ presented different profiles according to the presence of OA and the AP composition. All formulations showed CUR gradual permeation during the experiment (48 h), and no different profiles were observed until 12 h. However, from this time point, formulations were segregated in different profiles where higher CUR-permeated concentrations were obtained for the systems OA-PL F-127 and OA-PL F-127/L-81 than those determined for OA, PL F-127-H, and PL F-127/L-81-H. In fact, CUR permeation rate was significantly enhanced (p < 0.001) by OA-PL F-127/L-81 (4.83 µg.cm^−2^) compared with OA-PL F-127 (3.51 μg.cm^−2^), OA (2.25 μg.cm^−2^), PL F-127-H (1.16 μg.cm^−2^), and PL F-127/L-81-H (1.21 μg.cm^−2^) (**Figure 4**). Similar results were also observed after comparisons among the parameters drug flux and permeability coefficient for OA-PL F-127/L-81, which were significantly lower in relation to hydrogels (PL F-127-H and PL F-127/L-81-H) and OA-PL F-127, with p < 0.001 and p < 0.01, respectively (**Table 3**). On the other hand, latency times were very close for all formulations (from 3.14 to 3.66 h), and no statistical differences were observed, indicating a possible drug retention into the formulation.

### *In Vitro* Cytotoxicity and Antileishmanial Activity

**Figure 5:**
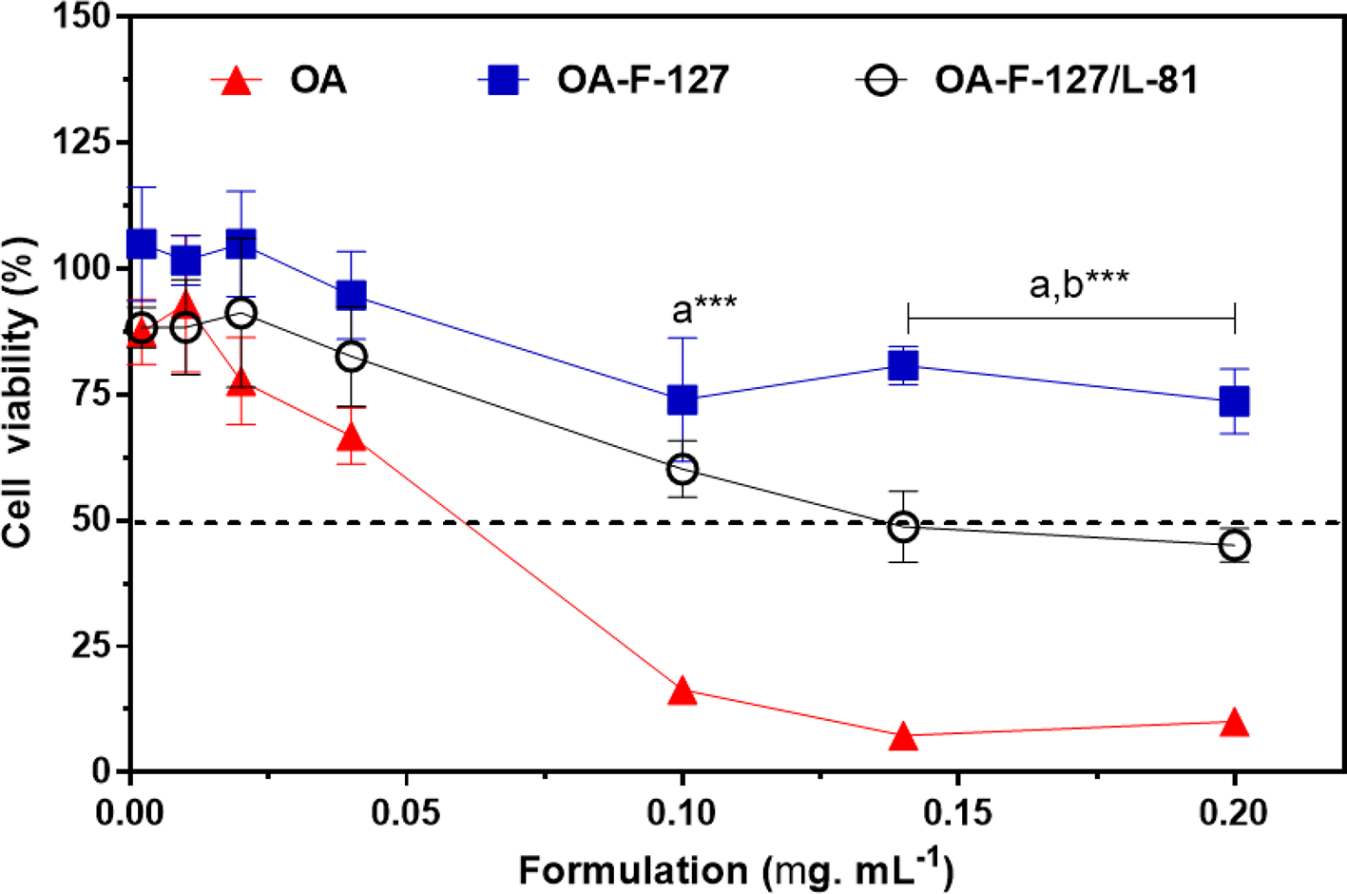
Effects of organogels formulations on HaCat cells determined by MTT reduction test. Data expressed as mean ± standard deviation with n = 6 replicates/concentration). F-127, Pluronic^®^ F-127; L-81, Pluronic^®^ L-81; OA, oleic acid. a- OA-F-127 and OA-F-127/L-81 *vs.* OA; b- OA-F-127 *vs.* OA-F-127/L-81. *** p < 0.001.

*In vitro* cytotoxicity assays were carried out in order to assess the effects of the vehicle OA, and the formulations (OA-PF F-127 and OA-PL F-127/L-81) in keratinocytes from HaCat cell line, evaluated by the MTT reduction test. All results are presented on **Figure 5**.

The increase on OA concentrations evoked toxic effects to the cells. However, the cell treatment with both ORG formulations did not induce pronounced cell toxicity. In addition, OA-PF F-127 presented the lowest cytotoxic effects (p < 0.001), with percentages ranging from 100 to 73.6%, compared to OA-PL F-127/L-81 (from 87.1 to 45.5%) and OA (from 87.4 to 10.1%), since the incorporation of OA into the PL-based AP reduced its cytotoxicity, being associated to the highest cell viability percentages. In addition, the reduced cell viability percentage after PL L-81 incorporation can be due to its lower HLB value compared to PL F-127, which possibly enhanced the PL cell membrane partitioning.

Additionally, the potential leishmanicidal activity was also investigated. All ORGs were incubated with *L. amazonensis* promastigote forms during 24h and their effects compared with amphotericin B, as positive control. In general, all ORG formulations OA, OA-PL F-127, and OA-PL F-127/L-81 evoked pronounced antileishmanial activity (IC_50_ < 1.25 µg.ml^−1^) before and after CUR incorporation. Additionally, it is necessary to highlight that all formulations presented lower IC values compared with isolated CUR (IC_50_ = 9.45 µg.ml^−1^), as observed on **Table 4**. Even considering that low CUR concentration can be permeated from ORGs, all formulations exhibited leishmanicidal effects suggesting their potential use as skin delivery systems against *L. amazonensis*.

**Table 4:** Percentage of inhibition of growth *L. amazonensis* promastigote forms/concentrations (µg.ml^−1^).

Treatments	Concentrations (µg/ml)					
	20	10	5.0	2.5	1.25	IC_50_
**OA** **OA-F-127**	100 ± 0.00	100 ± 0.00	100 ± 0.00	100 ± 0.00	100 ± 0.00	<1.25
**OA-F-127/L-81**						
**OA-F-127-CUR**						
**OA-F-127/L-81-CUR**						
**CUR**	74.00 ± 5.65	57.50 ± 3.53	19.50 ± 0.70	14.70 ± 3.53	8.20 ± 0.56	9.45 (8.00–11.24)#
	**0.40**	**0. 20**	**0.10**	**0.05**		**IC_50_**
**Amph. B**	66.57 ± 3.06	56.79 ± 2.99	40.50 ± 0.70	31.90÷0.14		0.15 (0.13–0.21)#

Results were expressed as the mean percentage of growth inhibition relative to the negative control. Two experiments were performed in triplicate. The 50% inhibitory concentration values (IC_50_) and 95% confidence limits (in parenthesis) were determined by non-linear regression curves. Positive control: amphotericin B, Amph. B; negative control: medium RPMI +0.1% dimethylsulfoxide. F-127, Pluronic® F-127; L-81, Pluronic® L-81; OA, oleic acid.

## Discussion

Natural products have been reported as a source of medicines for thousands of years. Since the discovery of pure compounds as bioactive molecules, the art of exploring natural products has become part of the molecular sciences. Many drugs used to treat different pathologies have been extracted from plants, and CUR has a long application for the treatment of several pathological processes including inflammatory, immunogenic, wound healing, and infectious conditions (Priyadarsini, 2014; de Moraes, 2015; Vaughn et al., 2016).

Despite its extensive pharmacological activities, CUR presents physico-chemical limitations, such as low aqueous solubility and bioavailability, reducing its permeation across the skin, since the clinical efficacy of bioactive molecules administered by topical route depends mainly on their physico-chemical and pharmacological properties, as well as their bioavailability at the site of action, which is limited by the low permeability of the stratum corneum. Then, due to the special structure and skin properties, novel formulations, such as lipid-based ORGs, have been developed in attempt to overcome those limitations.

In this study, we have presented the development, physico-chemical characterization, and biopharmaceutical evaluation of ORGs for CUR skin-delivery. In special, we designed the formulation compositions in order to carry high amounts of CUR soluble in OA (OP) and, then, entrapped into a tridimensional PL-based micellar AP, associating two polymers with different HLB values (PL F-127 and PL L-81) for promoting the permeation enhancement across the skin.

In this context, comparisons between the systems OA-PL F-127 and OA-PL F-127/L-81 suggest that the differences on thermodynamics parameters can be attributed to the presence of L-81 on AP. Since, PL L-81 is more hydrophobic (HLB = 8) compared to PL F-127 (HLB = 22), this feature can favor the possible CUR incorporation into micelle–micelle interface, as previously described by other hydrophobic drugs (Sharma et al, 2008; Esposito et al., 2018; Vigato et al., 2019). Additionally, those observations can suggest that different endothermic processes are capable to promote changes on ORG structural organization, particularly considering variations on aqueous and/or OP compositions.

As expected for hydrophobic molecules, such as CUR (log P = 3.62), there is a high partition on OP, but is possible that these molecules could be interacting with micellar central propylene glycol hydrophobic blocks from PL molecules, especially for the system composed by the association PL F-127/L-81. Additionally, it is necessary to point out CUR reduced both G’/G” relationship and η* values, as an indicative of a disturbance on the ORG network structural organization caused by the CUR molecule insertion into the system. Those results are also in agreement with previous reports describing the structural organization of PL-based systems for delivering hydrophobic molecules (Sharma et al., 2008; Sharma et al., 2018; Nascimento et al., 2018; Vigato et al., 2019), also corroborating the calorimetric results. Although this effect has been observed, ORG formulations maintained the high G’/G” ratio, an important feature to obtain adequate spreadability, forming a thin film on skin, but without loss of the formulation structural organization and potentially prolonging the contact time with the skin.

Despite the differences between *in vitro* (using artificial membranes) and *ex vivo* skin permeation profiles, Strat-M^®^ has been used as skin model for evaluating the drug diffusion profiles from new delivery systems during the early stages of the development. In fact, its lipid matrix composition (ceramides, free fatty acids, cholesterol, and phospholipids) can simulate the skin barrier, being useful for determining permeation parameters for different types of pharmaceutical formulations such as hydrogels, nanoparticulate systems, emulsions, and ORGs (Uchida et al., 2015; Simon et al., 2016; Haq et al., 2018; Grillo et al., 2019; Vigato et al., 2019).

Several studies have been reported regarding the development of new skin delivery systems for CUR such as monoolein aqueous dispersion and lecithin ORGs (Esposito et al., 2014), liquid crystalline systems composed of OA, polyoxypropylene/polyoxyethylene cetyl alcohol (Fonseca-Santos et al., 2016), Pluronic F-127/P-123 micelles (Akbar et al., 2018), Pluronic F-127 hydrogel (Yen et al., 2018), and methoxy poly (ethylene glycol)-block-poly (ε-caprolactone) (MPEG-PCL) hydrogels (Zhou et al., 2019) for antioxidant, anti-inflammatory, antileishmanial, and wound-healing purposes. However, the influence of structural parameters on drug permeation, the presence of OA, and association of polymers on AP have been not discussed. In this context, we can postulate that the high permeated CUR amounts from the system OA-PL F-127/L-81 can be attributed to some structural factors: (i) the hydrophobic interactions between CUR and OA, into the OP, promoting the drug solubilization and acting as a permeation enhancer; (ii) the association of L-81 into the AP that, possibly, allowed the CUR interaction with its PPO hydrophobic units on organic-AP interface and also promoted CUR incorporation into Strat-M^®^ lipid matrix; and (iii) the formation of a more fluid ORG system, OA-PL F-127/L-81, presenting lower viscosity and G’/G” relationship, compared to OA-PL F-127, but capable to maintain the formulation in contact with the skin-membrane model and enhance the drug permeation. Additionally, different PL-based systems have been used as new therapeutic strategies for several purposes, such as leishmaniosis treatment. Recent reports described formulations based on PL F-127 micelles encapsulating a naphthoquinone derivative (Mendonça et al., 2019), clioquinol (Tavares et al., 2019), amphotericin B (Mendonça et al., 2016), and PL F-68 micellar systems for amphotericin B (Espuelas et al., 2000). In this study, we present the development of lipid-PL formulations containing CUR; in particular, OA has been described as an important component of skin formulations for enhanced efficacy on leishmaniosis treatment (Pinheiro et al., 2016), as well as due to its involvement on transition from promastigotes to amastigotes (Bouazizi-Ben et al., 2017). In this context, further experiments will be necessary in order to evaluate the performance of each formulation and its isolated components on proliferation process for both promastigote and amastigote host-cell stages as well as on different *Leishmania* strains. In summary, results from this study pointed out OA-PL-based ORGs as promising new formulations for CUR skin delivery with potential pharmacological activity against *L. amazonensis*.

## Author Contributions

AV, NF, SQ and IM were responsible for physico-chemical characterization experiments, summarized the data and wrote the manuscript. EC and LF performed microscopy analysis. CC and GT were responsible for cell culture assays. AC and LC carried out antileishmanial activity assays. MS and DA contributed to the design, review and wrote the manuscript.

## Funding

This research work was supported by Coordenação de Aperfeiçoamento de Pessoal de Nível Superior (CAPES), Fundação de Amparo à Pesquisa do Estado de São Paulo (FAPESP 2014/14457-5, 2018/04036-3, 2018/02482-6, 2016/18045-9, 2016/24456-1), Conselho Nacional de Desenvolvimento Científico e Tecnológico (CNPq 309207/2016-9, 402838/2016-5, 303946/2018-0) and UFABC Multiuser Central Facilities (CEM-UFABC).

## Conflict of Interest Statement

The authors declare that the research was conducted in the absence of any commercial or financial relationships that could be construed as a potential conflict of interest.
